# Accumulation of Cytotoxic Skin Resident Memory T Cells and Increased Expression of IL-15 in Lesional Skin of Polymorphic Light Eruption

**DOI:** 10.3389/fmed.2022.908047

**Published:** 2022-06-10

**Authors:** VijayKumar Patra, Johanna Strobl, Denise Atzmüller, Bärbel Reininger, Lisa Kleissl, Alexandra Gruber-Wackernagel, Jean-Francois Nicolas, Georg Stary, Marc Vocanson, Peter Wolf

**Affiliations:** ^1^Centre International de Recherche en Infectiologie, Institut National de la Santé et de la Recherche Médicale, U1111, Université Claude Bernard Lyon 1, Centre National de la Recherche Scientifique, UMR 5308, Ecole Normale Supérieure de Lyon, Université de Lyon, Lyon, France; ^2^Research Unit for Photodermatology, Medical University of Graz, Graz, Austria; ^3^Department of Dermatology, Medical University of Vienna, Vienna, Austria; ^4^Ludwig Boltzmann Institute for Rare and Undiagnosed Diseases, Vienna, Austria; ^5^Allergy and Clinical Immunology Department, Lyon Sud University Hospital, Pierre-Bénite, France; ^6^CeMM Research Center for Molecular Medicine of the Austrian Academy of Sciences, Vienna, Austria

**Keywords:** sun allergy, polymorphic light eruption, disease recurrence, inflammation, disease memory, resident memory T cell (TrM)

## Abstract

Patients with polymorphic light eruption (PLE) develop lesions upon the first exposure to sun in spring/summer, but lesions usually subside during season due to the natural (or medical) photohardening. However, these lesions tend to reappear the following year and continue to do so in most patients, suggesting the presence of a disease memory. To study the potential role of skin resident memory T cells (Trm), we investigated the functional phenotype of Trm and the expression of IL-15 in PLE. IL-15 is known to drive Trm proliferation and survival. Multiplex immunofluorescence was used to quantify the expression of CD3, CD4, CD8, CD69, CD103, CD49a, CD11b, CD11c, CD68, granzyme B (GzmB), interferon-gamma (IFN-γ), and IL-15 in formalin-fixed, paraffin-embedded lesional skin samples from PLE patients and healthy skin from control subjects. Unlike the constitutive T cell population in healthy skin, a massive infiltration of T cells in the dermis and epidermis was observed in PLE, and the majority of these belonged to CD8^+^ T cells which express Trm markers (CD69, CD103, CD49a) and produced cytotoxic effector molecules GzmB and IFN-γ. Higher numbers of CD3^+^ T cells and CD11b^+^CD68^+^ macrophages produced IL-15 in the dermis as compared to healthy skin. The dominant accumulation of cytotoxic Trm cells and increased expression of IL-15 in lesional skin of PLE patients strongly indicates the potential role of skin Trm cells in the disease manifestation and recurrence.

## Introduction

Polymorphic light eruption (PLE) is the most common form of immune-mediated photodermatosis, affecting around 10-20% of the young female population (1). It is considered to result from an immunological disturbance and breakdown in tolerance to ultraviolet radiation (UVR) (2–8). PLE patients develop pruritic and non-scarring lesions, which are associated with increased IL-31 (9) expression, and IL-1 family pro-inflammatory cytokines (10), altered clearance of apoptotic cells (11) and linked T cell infiltration, as early as few hours to days after first being exposed to sunlight (UVA and UVB), i.e., usually in early spring or summer (12). PLE occurs intermittently after sun exposure on the exposed body areas that are normally covered during winter, such as the upper chest, neck, and arms (13). These lesions usually subside within few days when sunlight exposure is avoided. As the summer progresses, PLE patients may experience a natural hardening of the skin, most likely due to photoimmunological mechanisms (14–16). This often results in a reduced likeliness of developing lesions and/or less severe clinical manifestations (17, 18). However, this photohardening (natural and/or artificial) effect is lost during winter, and PLE patients develop lesions again the next year, continuing to do so for many years to come (19, 20). This indicates that a specific disease memory is formed during the active stage of the disease (21), and that such a disease memory might be retained by the skin, favoring the recurrence of the disease.

Human skin is inhabited and constantly patrolled by large numbers of tissue-resident memory T (Trm) cells, which are associated with disease memory. Of the estimated 20 billion T cells contained in human skin, 50–70% of these express the Trm markers CD69 and CD103, which are responsible for CD4^+^ or CD8^+^ Trm cell lodging in epithelial tissues (22). Trm cells present in the skin display a potential for tolerance and immunity that is in dependent on the local microenvironment. Besides maintaining immunity, pathogenic Trm cells have been implicated in several inflammatory skin diseases, such as psoriasis, vitiligo, eczemas, and fixed drug eruption, among others (21). CD49a, constituting the α-subunit of the α1β1 integrin receptor [also known as very late antigen 1 (VLA-1)], binds collagen IV in the basement membrane that separates the epidermis from the dermis. The CD49a expression (about 15%) (23) on skin-derived T cells differentiates CD8^+^ Trm cells on a compartmental and functional basis and indicates their cytotoxic capacity, which might play putative roles in various cutaneous immunopathologies (24–27). In healthy skin, CD8^+^CD103^±^CD49a^–^ Trm cells are present in the epidermis and dermis, whereas CD8^+^CD103^+^CD49a^+^ Trm cells are localized in the epidermis (25). Upon IL-15 stimulation, the epidermal CD8^+^CD103^+^CD49a^+^ Trm cells produce perforin and GzmB, thereby inducing a strong and rapid cytotoxic response (25). The lesional skin of psoriasis patients features an accumulation of CD8^+^CD103^+^CD49a^–^ Trm cells expressing IL-17, whereas vitiligo skin shows a predominance of cytotoxic CD8^+^CD103^+^CD49a^+^ cells producing IFN-γ, perforin, and GzmB (25, 28). Acute UV exposure is known to rapidly activate skin Trm cells in both humans (with increased CD69 expression) and in mice (29), as well as to induce the expression of IL-15 (30). This suggests that UV exposure can act as external trigger to activate Trm cells directly or via the induction of IL-15 (31).

We hypothesized that PLE patients accumulate *in situ* skin resident memory T cells that might play a role in disease manifestation and particularly the disease recurrence upon UV exposure. To test this hypothesis, we used archived samples from lesional PLE skin and healthy skin from control subjects and performed multiplex immunofluorescence staining to characterize Trm cell subsets, and to assess their potential cytotoxicity. We found that 70–80% of the CD3^+^ T cells infiltrating the PLE lesions expressed the Trm markers CD69 and CD103. Intriguingly, significant amounts of the effector molecules GzmB and IFN-γ were expressed by these Trm cells. Furthermore, PLE lesions exhibited higher levels of IL-15 expression as compared to healthy skin, and the majority of the IL-15 was predominantly produced by CD3^+^ T cells and CD11b^+^ CD68^+^ macrophages. Taken together, our results reveal the infiltration of distinctly cytotoxic (Gzmb^+^ and IFN-γ^+^) Trm cells and the enhanced expression of IL-15 in PLE, two factors that are believed to synergistically play a key role in disease manifestation and recurrence.

## Materials and Methods

### Patients and Skin Biopsies

Formalin-fixed, paraffin-embedded lesional skin samples of 13 PLE patients (12 artificially photo-provoked and one exposed to natural sunlight) and 10 samples of healthy skin were available from the biobank at the Department of Dermatology (Medical University of Graz, Austria) for investigations. The investigational studies were approved by the Ethics Committee of the Medical University of Graz, Austria (18-068 ex 06/07 and 25-293 ex 12/13). The work was performed following the guidelines of the Declaration of Helsinki Principles. Detailed information about the patient and sample characteristics is found in our previous publications (9, 32).

### Immunofluorescence

Multiplex immunofluorescence staining was performed on formalin-fixed paraffin-embedded tissue sections. In brief, slides were de-paraffinized by baking them at 80°C, followed by Neo-Clear and ethanol sequences, and heat-induced antigen retrieval. Subsequently, slides were blocked using mouse serum and stained with directly and indirectly labeled monoclonal antibodies in multiple steps, including overnight incubation. Isotype-matched control antibodies were stained simultaneously. Finally, counterstaining was performed with 4’,6-diamidino-2-phenylindol (DAPI). Detailed information about the antibodies used in this study are provided in the Supplementary Material.

### Image Analysis

The image analysis was performed using a TissueGnostics (Vienna, Austria) imaging system as previously described (33). Images of tissue sections were acquired using a Z1 Axio Observer microscope equipped with a LD Plan-Neofluar 20x/0.4 objective (Zeiss) and TissueFAXS software. Epidermal and dermal regions were manually selected followed by automated cell quantification using the TissueQuest image analysis software and manual correction.

### Statistical Analysis

We performed statistical analyses in GraphPad Prism, Version 9.3 (GraphPad Software). The level of statistical significance was determined by applying the Mann–Whitney test. *P*-values of less than 0.05 were deemed statistically significant.

## Results

### CD49a^+^ Trm Cells Are Enriched in Active PLE Lesions

**FIGURE 1:**
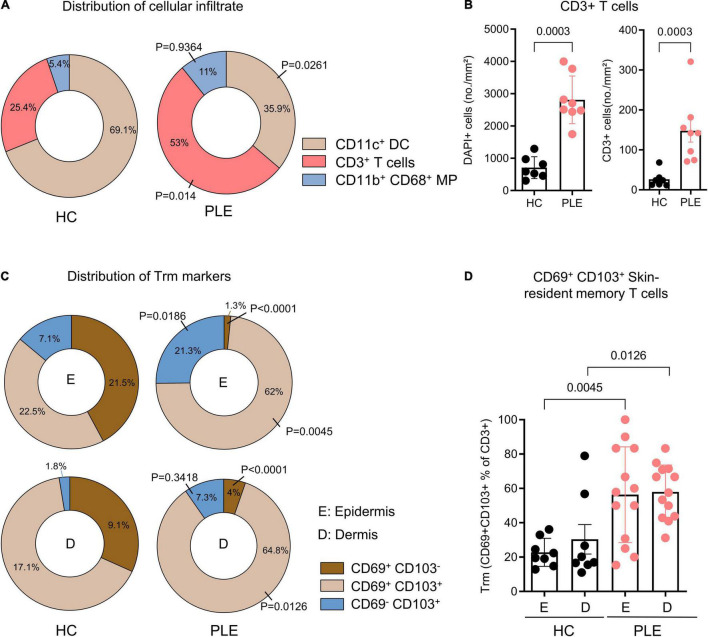
Increased percentage of CD69^+^ CD103^+^ skin Trm cells in PLE lesions. **(A)** Relative distribution of CD11c^+^ dendritic cell (DC), CD3^+^ T cells, and CD11b^+^ CD68^+^ macrophages (MP) in healthy control (HC) and polymorphic light eruption (PLE), number shown as mean percentage of all categorized cells. Statistical analysis with Mann–Whitney test **(B)** Number of DAPI^+^ cells and CD3^+^ T cells in healthy control (HC) and polymorphic light eruption (PLE) skin. Data shown as a mean cell number/mm^2^ ± SD (HC: *n* = 7, PLE: *n* = 8). **(C)** Distribution of cells positive for Trm surface markers on CD3^+^ T cells of the epidermis (E) and dermis (D) from immunostaining data. Data are shown as a relative mean percentage of all samples in the pie charts. **(D)** Tissue residency marker-expressing T cells in PLE and healthy control skin; data shown as a percentage of CD69^+^ and CD103^+^ T cells among total CD3^+^ T cells ± SD. Statistical analysis with Mann-Whitney test, P values for PLE vs HC are indicated on the graphs (HC: *n* = 8, PLE: *n* = 13).

**FIGURE 2:**
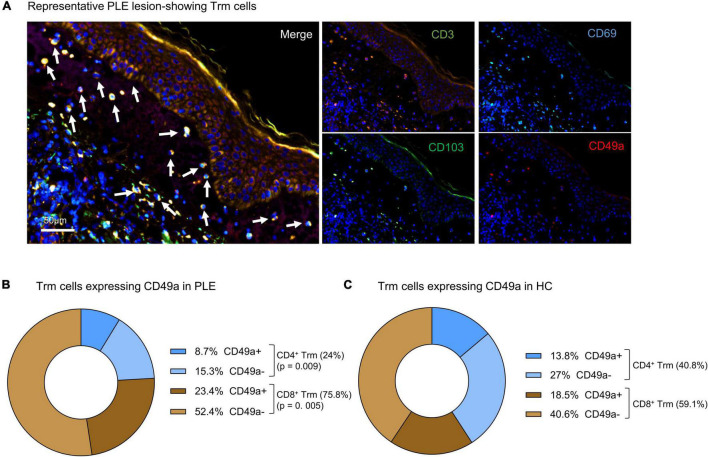
Skin Trm cells of PLE lesions express CD49a. **(A)** Representative image of immunofluorescent staining of a PLE section; arrows indicate CD3^+^CD69^+^CD103^+^CD49a^+^ Trm cells localized in the dermis and near the dermo-epidermal junction. **(B)** Percentage of CD4^+^ or CD8^+^ tissue residency marker distribution on T cells in PLE lesions and in panel **(C)** HC skin expressing CD49a is shown in the pie charts; data shown as a mean percentage of all tissue-resident T cells from immunostaining data. Statistical analysis with Mann–Whitney test, (HC: *n* = 11, PLE: *n* = 13).

To characterize the cellular infiltrate such as T cells (CD3^+^), dendritic cells (CD11c^+^CD11b^–^), and macrophages (CD11b^+^CD68^+^) in PLE, we performed multiplex IF staining of the patient’s samples (Figures 1A,B). Among the total T cells (CD3^+^) infiltrating the lesional skin of PLE (148 ± 80.2 vs 26.4 ± 19.9 cells/mm^2^ in HC) (Figure 1B), most cells expressed the key tissue residency markers CD69 (sphingosine-1-phosphate suppressor) and CD103 (integrin αE) (Figure 1D). Overall, the numbers of CD69^+^ CD103^+^ Trm cells appeared to be significantly increased in both the epidermis (56.3 ± 27.8 cells/mm^2^) and dermis (57.9 ± 15.6 cells/mm^2^) in PLE as compared to healthy skin (epidermis: 22.8 ± 8.1; dermis: 30.3 ± 24.3 cells/mm^2^) (Figures 1C,D). To gain further insight into the diversity of Trm cells with respect to spatial niches, we performed staining to identify the Trm-associated markers CD69, CD103, and CD49a on CD4^+^ and CD8^+^ T cells (Figure 2A). In lesional skin of PLE, most Trm cells were CD8^+^ (75.8%) rather than CD4^+^ (24%), and among the CD8^+^ Trm cells, a large proportion of cells expressed CD49a (23.4%) (Figure 2B). In contrast, the proportions of CD4^+^ (40.8%) and CD8^+^ (59.1%) T cells expressing Trm markers were more balanced in healthy skin. However, unlike PLE, healthy skin contained higher percentages of CD4^+^ (27%) and CD8^+^ (40.6%) Trm cells that are CD49a^–^ (Figure 2C). Taken together, these data demonstrate the impressive proliferation of CD49a^+^ Trm cells in PLE.

### Skin Trm Cells in PLE Lesions Express Increased Levels of Effector Cytotoxic Molecules

**FIGURE 3:**
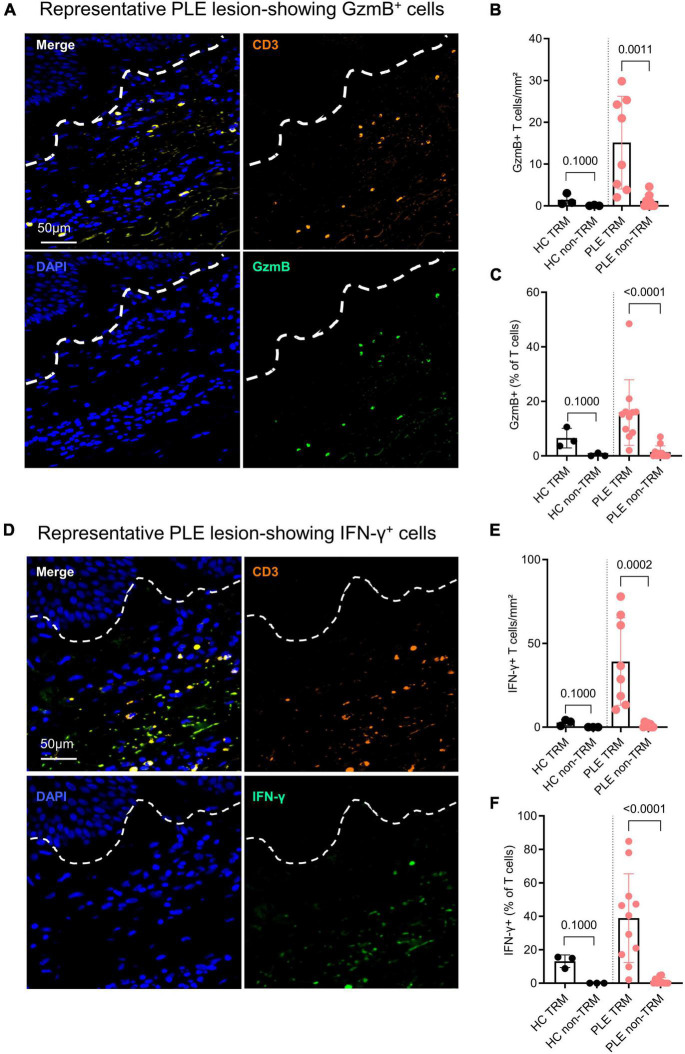
Trm cells show high inflammatory cytokine production in active PLE lesions. **(A,D)** Representative IF images of granzyme B (GzmB) and interferon gamma (IFN-γ) staining in a PLE lesion; dotted line indicates dermo-epidermal border. Production of GzmB **(B)** and IFN-γ **(E)** by T cells in PLE skin as compared to healthy control (HC) skin. Data shown as a mean number of positively stained CD3^+^ T cells/mm^2^ ± SD. **(C,F)** Production of GzmB **(C)** and IFN-γ **(F)** by TRM (CD3^+^ CD69^+^) as compared to non-TRM (CD3^+^ CD69^–^) T cells. Data shown as a percentage of T cells ± SD positive for GzmB or IFN-γ. Statistical analysis with Mann–Whitney test (HC: *n* = 3, PLE: *n* = 8).

IFN-γ and GzmB are key effector cytokines released by cytotoxic T cells that play a role in various inflammatory skin disorders (21). To assess the cytotoxic nature of the Trm cells in PLE, we performed IF staining to detect GzmB (Figure 3A) and IFN-γ (Figure 3D). As compared to healthy skin, lesional skin of PLE showed significantly increased levels of GzmB- and IFN-γ-producing CD3^+^ T cells, mostly in the dermis (Figure 3B). Significantly more GzmB and IFN-γ was produced by CD3^+^ CD69^+^ Trm cells (GzmB:15.1 ± 11.1; IFN- γ: 39.2 ± 26.1 cells/mm^2^) than by CD3^+^ CD69^–^ T cells (“non-Trm”) (GzmB: 1.1 ± 1.6; IFN- γ: 1 ± 1.2 cells/mm^2^) in the lesional skin of PLE patients compared to healthy skin (Figure 3). Healthy skin showed little or no expression of the cytotoxic cytokines by CD3^+^CD69^+^ Trm cells (GzmB: 1.4 ± 1.3; IFN- γ: 2.7 ± 1.7 cells/mm^2^) or non-Trm (CD3^+^CD69^–^) cells. Taken together, these results indicate that the Trm cells in healthy skin appear to be in a state of quiescence, whereas the Trm cells in an inflammatory condition like PLE appear to exhibit cytotoxic activity by producing GzmB and IFN- γ.

### Increased Expression of IL-15 by T Cells and Macrophages in PLE

**FIGURE 4:**
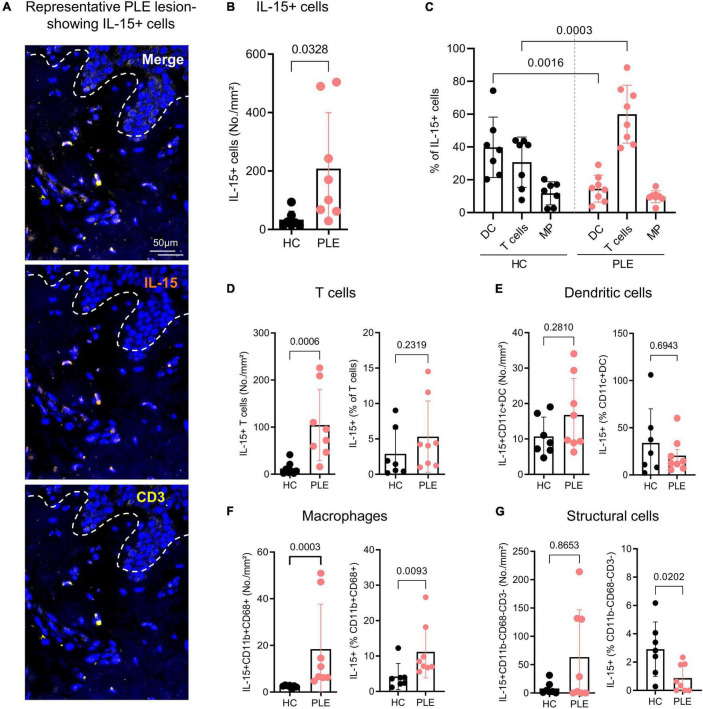
CD3^+^ T cells and CD68^+^ macrophages are the major cellular source of IL-15 in PLE. **(A)** Representative immunofluorescent image of IL-15^+^ CD3^+^ T cells in PLE; dotted line indicates the dermo-epidermal border. **(B)** Number of IL-15-producing DAPI^+^ cells in healthy control (HC) skin and polymorphic light eruption (PLE) lesions. Data shown as a mean cell number/mm^2^ ± SD. **(C)** Cellular source of IL-15 in PLE; data shown as a percentage of dendritic cell (DC), T cells, and macrophages (MP) of total IL-15^+^ cells ± SD. **(D–G)** Mean number and percentage of IL-15-producing CD3^+^ T cells **(D)**, CD11c^+^ dendritic cells **(E)**, CD11b^+^ CD68^+^ macrophages **(F)** and CD11b^–^ CD68^–^ CD3^–^ structural cells **(G)** per mm^2^ ± SD in HC skin and PLE. Statistical analysis with Mann–Whitney test, (HC: *n* = 7, PLE: *n* = 8).

IL-15 is a key cytokine that preferentially stimulates CD8^+^ T cell activation, proliferation, and cytolytic activity; it is required for Trm maintenance (34–37). Previous research has shown that IL-15 stimulation also strongly prompts cellular cytotoxicity in CD8^+^ CD69^+^CD103^+^CD49a^+^ Trm cells in a TCR-dependent manner (25). Having found that Trm cells in PLE lesions express unusually high amounts of cytotoxic markers, we set out to investigate IL-15 expression in our samples and found that PLE lesions (208.4 ± 190.6 cells/mm^2^) had increased levels of IL-15^+^ cells as compared to healthy skin (33.9 ± 28.9 cells/mm^2^) (Figures 4A,B). To further address which cells produced IL-15 in PLE lesions, we performed multiplex IF staining for T cells (CD3^+^), dendritic cells (CD11c^+^), macrophages (CD11b^+^ CD68^+^), and structural cells (DAPI^+^CD3^–^CD11b^–^CD68^–^) (Figure 4C–G). We observed that T cells (104.3 ± 75.6 cells/mm^2^) and macrophages (18.4 ± 19.2 cells/mm^2^) primarily produce IL-15 in PLE lesions, while dendritic cells (16.7 ± 10.3 cells/mm^2^) produce IL-15 to a lesser extent (Figures 4D–F) compared to healthy skin (T cells: 11.78 ± 13.4; macrophages: 2.4 ± 0.6; dendritic cells: 10.7 ± 5.4 cells/mm^2^). CD3^–^CD11b^–^CD68^–^ structural cells, which include keratinocytes and low-populated immune cells, expressed IL-15 in certain PLE samples (63.4 ± 83.4 cells/mm^2^) but showed little or no expression in healthy skin (8 ± 11.3 cells/mm^2^) (Figure 4G). Thus, an increased production of IL-15 by T cells and macrophages was found in PLE lesions.

## Discussion

Resident memory T (Trm) cells have been associated with multiple cutaneous inflammatory diseases, and their role in disease manifestation and recurrence in psoriasis, vitiligo, eczemas, and fixed drug eruption has been addressed (21, 28, 38–40). Patients suffering from PLE experience peculiar recurrences of the disease after sun exposure every year in spring or summer. In fact, results of a recent registry analysis of PLE patients indicate that, out of 97 patients analyzed, 74% of the patients still suffered from PLE 20 years after the first eruption, with the lesions often persisting for more than a week during disease episodes (19). However, the pathophysiology behind the prolonged course of the disease has remained unknown. The results of this study indicate that PLE lesions are infiltrated by a large number of CD3^+^ T cells, the majority of which express the tissue residency markers CD69 and CD103. These Trm cells are localized in both the epidermis and dermis. In psoriasis and vitiligo, Trm cells expressing CD49a as a subpopulation of CD8^+^ Trm cells specifically localize in the basal layers of epidermis and, upon stimulation by IL-15, preferentially produce IFN-γ and display a high cytotoxic capacity (25). Similarly, we identified skin Trm cells that were localized in the basal layers of epidermis and dermis in PLE lesions. Among CD8^+^ Trm cells in PLE lesions, the majority expressed CD49a, suggesting that this Trm marker has cytotoxic function. This hypothesis was further supported by the fact that Trm cells primarily produced the effector molecules GzmB and IFN-γ in PLE. These results indicate that the observed abundance of cytotoxic Trm cells in the skin of PLE might favor disease manifestation and recurrence.

Skin Trm cells are known to gain cytotoxic capacity upon stimulation by IL-15 (25). When CD8^+^ CD103^+^CD49a^+^ Trm cells were stimulated *in vitro* by IL-15, they strongly produced effector cytotoxic molecules such as GzmB (25). This increased IL-15 production might be of functional relevance in PLE. Indeed, IL-15 is known not as a constitutive cytokine that is expressed by keratinocytes, but rather as a cytokine induced by external stimuli or during inflammation (41). Intriguingly, when human skin is exposed to UVB, a higher amount of IL-15 is expressed by keratinocytes, both at the mRNA and protein level (30). Apart from keratinocytes, DCs, monocytes/macrophages, bone marrow stromal cells, fibroblasts, and T cells can also produce IL-15. Interestingly, T cell-produced, functionally active IL-15 is able to induce T cell proliferation in an autocrine/juxtracrine loop (42). Most IL-15 in PLE is produced by CD3^+^ T cells and CD68^+^ macrophages and, to a lesser degree by DCs and keratinocytes. Interestingly, these macrophages are known to be major producers of the itch cytokine IL-31 in PLE lesions; thus, they are considered to contribute to itch (9). The exact mechanism triggering the production of IL-15 in PLE remains to be determined, although previously published data underline the role of UVB in inducing IL-15 in human skin, which might initiate the development of PLE. We have previously hypothesized that skin microbiome or microbial elements may play a role in the pathogenesis of PLE (43). Intriguingly, in this regard, IL-15 has been known to modulate microbial communities (44); therefore, it is plausible that a high IL-15 expression might alter the microbiome in PLE. This can lead to the generation of microbe- and/or microbial antigen-specific skin Trm, and the (re)activation of such cells, therefore, could have broader implications, e.g., launching inappropriate responses against external antigens and contributing to the chronicity of the disease (45, 46). Moreover, IL-15 is known to induce macrophage differentiation and trigger cathelicidin peptide (LL-37) (47). In this regard, we have previously reported an increased expression of LL-37 in PLE lesions and enhanced macrophage infiltration (9, 32). Taken together, these results indicate that IL-15 may play a complex role in PLE; further work is warranted to investigate the rather complex interplay between UVR, the skin microbiome, IL-15, and skin Trm cells (31).

As PLE lesions are known to subside within a few days if exposure to sunlight is avoided, the state of Trm cell activation still needs to be investigated. Although our results show that Trm cells in PLE are in an activated cytotoxic state, previous reports of Trm cells in gut epithelia have indicated that the activation state of Trm cells might be time limited (48). A transient state of Trm activation could also explain why PLE lesions subside overtime. Intriguingly, PLE patients exhibit low regulatory T (Treg) cell numbers early on in the season, and these numbers increase as the season advances (49, 50). Moreover, photohardening with narrowband-UVB increased the functional capacity of Tregs in PLE (49, 51, 52). It is interesting to note that Tregs can affect other T cells, and especially CD8^+^ T cells, during the effector stage and reduce their functional abilities (53, 54). Moreover, Tregs can also enhance Trm cell evolution and initiate their arrest in a semi-activated state (55). Unlike other T cell subsets, Trm cells are extremely resistant to X-ray radiation, and medical photo-hardening treatment given to PLE patients at suberythemal doses might be much too inefficient at killing those cells or making any other direct impact on them (56). That said, photo hardening has been shown to significantly increase mast cell numbers in PLE patients (17), and another study reported that mast cells can consume large amounts of IL-15 and control the accumulation of CD8^+^ Trm cells (57). Therefore, a hardening effect in PLE might result from the influence of UV on Tregs and mast cells, which, in turn, may downregulate or inhibit Trm cell activity.

The limitations of this study are its overall small sample size and the imperfect patient age and sex match. Furthermore, as no healthy, non-lesional skin from PLE patients was available to be examined in this study, we cannot draw conclusions about the state of Trm cells in the skin during the PLE-free intervals between disease episodes. Future studies need to investigate whether the induction and maintenance of Trm in PLE is site-specific. Nevertheless, our findings of an increased accumulation of cytotoxic Trm cells, linked to an increased IL-15 expression, clearly indicates that skin Trm cells play active roles in the disease manifestation and recurrence of PLE. This may open avenues for the development of novel treatment strategies in PLE, directed at selectively eliminating skin Trm cells. In particular, IL-15 could also serve as a potential target for preventing PLE and/or treating PLE patients. Indeed, anti-IL-15 blockade in psoriasis (58) and vitiligo (59) has been investigated as a promising therapeutic concept in those diseases.

## Data Availability Statement

The raw data supporting the conclusions of this article will be made available by the authors, without undue reservation.

## Ethics Statement

The studies involving human participants were reviewed and approved by Ethics Committee of the Medical University of Graz, Austria. The patients/participants provided their written informed consent to participate in this study.

## Author Contributions

VP conceptualized the ideas, analyzed the data, drafted the manuscript, and prepared the figures. JS designed experiments, performed the stainings, analyzed the data, generated the figures, and contributed to drafting the manuscript. DA conducted the experiments and analyzed the imaging data. AG-W and PW acquired the patients’ samples. PW supervised the study. Co-first authors can prioritize their names when adding this manuscript reference to their resumes. All authors contributed to interpretation of the data and critically revised the manuscript to provide important intellectual content.

## Conflict of Interest

The authors declare that the research was conducted in the absence of any commercial or financial relationships that could be construed as a potential conflict of interest.

## Publisher’s Note

All claims expressed in this article are solely those of the authors and do not necessarily represent those of their affiliated organizations, or those of the publisher, the editors and the reviewers. Any product that may be evaluated in this article, or claim that may be made by its manufacturer, is not guaranteed or endorsed by the publisher.
