# Supplementary material for: Accumulation of Cytotoxic Skin Resident Memory T Cells and Increased Expression of IL-15 in Lesional Skin of Polymorphic Light Eruption
Source: Front Med (Lausanne). 2022 Jun 10;9:908047. doi: 10.3389/fmed.2022.908047 (PMC9226321; doi:10.3389/fmed.2022.908047)
Supplement: Supplementary file 1 [file Table_1.DOCX]

**Supplementary information**

**Supplementary table 1: Antibody list**

| **Antibody** | **Supplier** | **Catalogue No.** | **Clone** |
| --- | --- | --- | --- |
| CD103 | Biolegend | 350206 | Ber-ACT8 |
| CD11b | Biolegend | 101235 | M1/70 |
| CD11c | Biolegend | 301634 | 3.9 |
| CD11c | Biolegend | 337214 | Bu15 |
| CD3 | Biolegend | 300449 | UCHT1 |
| CD3 | BD | 345767 | SK7 |
| CD4 | BD | 345768 | SK3 |
| CD49a | BD | 559596 | SR84 |
| CD68 | Biolegend | 333809 | Y1/82A |
| CD69 | BD | 555530 | FN50 |
| CD69 | Biolegend | 310930 | FN50 |
| CD8 | Biolegend | 300912 | HIT8a |
| CD8a | R&D | FAB1509S | 37006 |
| Granzyme B | Sanquin Amsterdam | M2289 | GB11 |
| IFN-g | BD | 340449 | 25723.11 |
| IL-15 | R&D | IC2471T-100UG | 34559 |
