# Supplementary figures and images for: Accumulation of Cytotoxic Skin Resident Memory T Cells and Increased Expression of IL-15 in Lesional Skin of Polymorphic Light Eruption
Source: Front Med (Lausanne). 2022 Jun 10;9:908047. doi: 10.3389/fmed.2022.908047 (PMC9226321; doi:10.3389/fmed.2022.908047)

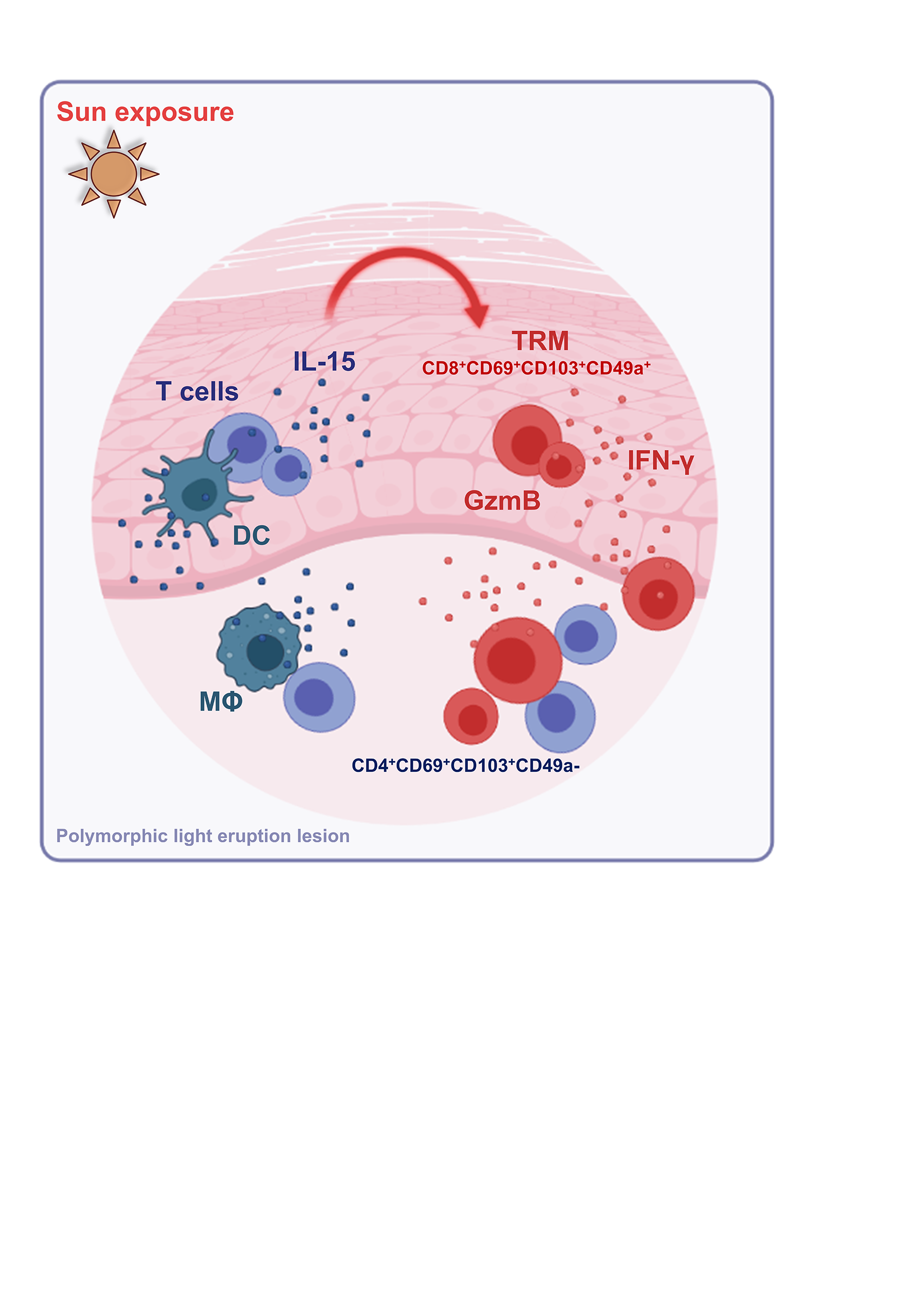

Supplement: Supplementary file 2 [file Image_1.TIF]
